# Supplementary material for: Slow light in a 2D semiconductor plasmonic structure
Source: Nat Commun. 2022 Oct 20;13:6216. doi: 10.1038/s41467-022-33965-8 (PMC9585030; doi:10.1038/s41467-022-33965-8)
Supplement: Supplementary file 1 — Supplementary Information [file 41467_2022_33965_MOESM1_ESM.pdf]

Supplementary Information for

Slow Light in a 2D Semiconductor Plasmonic Structure

Author Names: Matthew Klein[1], Rolf Binder[1,2] Michael R. Koehler[3], David G. Mandrus[3-5], Takashi Taniguchi[6], Kenji Watanabe[7], and John R. Schaibley[1]

Author Addresses:

[1]Department of Physics, University of Arizona, Tucson, Arizona 85721, USA

[2]Wyant College of Optical Sciences, University of Arizona, Tucson, Arizona 85721, USA

[3]Department of Materials Science and Engineering, University of Tennessee, Knoxville, Tennessee, 37996, USA

[4]Materials Science and Technology Division, Oak Ridge National Laboratory, Oak Ridge, Tennessee, 37831, USA

[5]Department of Physics and Astronomy, University of Tennessee, Knoxville, Tennessee, 37996, USA

[6]International Center for Materials Nanoarchitectonics, National Institute for Materials Science, 1-1 Namiki, Tsukuba 305-0044, Japan

[7]Research Center for Functional Materials, National Institute for Materials Science, 1-1 Namiki, Tsukuba 305-0044, Japan

Corresponding Author: John Schaibley, johnschaibley@arizona.edu

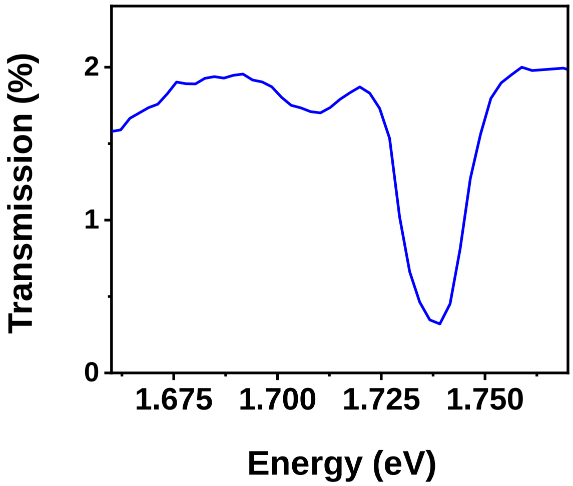

Supplementary Fig. 1: Linear transmission of the 2D semiconductor plasmonic structure.

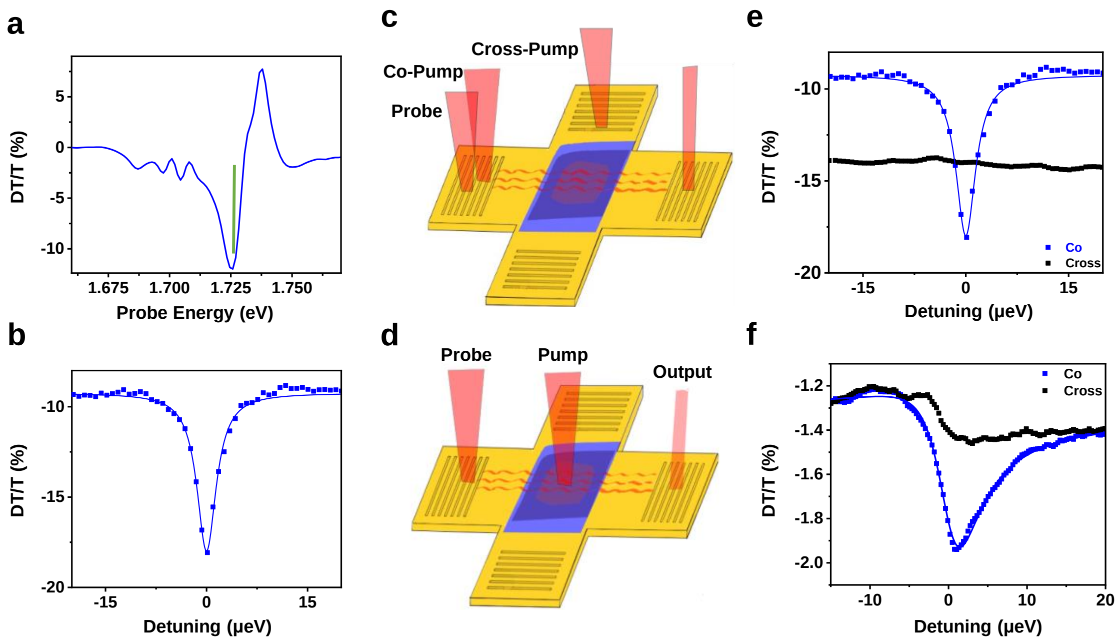

Supplementary Fig. 2: CPO on a second device. a, Broad DT/T for co-propagating SPP pump-SPP probe. The green bar depicts the pump position. **b**, High resolution DT/T spectra for co-propagating SPPs being pumped at 1.724 eV (719 nm). **c**, Depiction of the SPP pump-SPP probe configuration. **d**, Depiction of the optical pump-SPP probe configuration. **e-f**, High resolution DT/T spectra for SPP pump-SPP probe (optical pump-SPP probe). The blue points correspond to co-polarized, and the black correspond to cross-polarized.

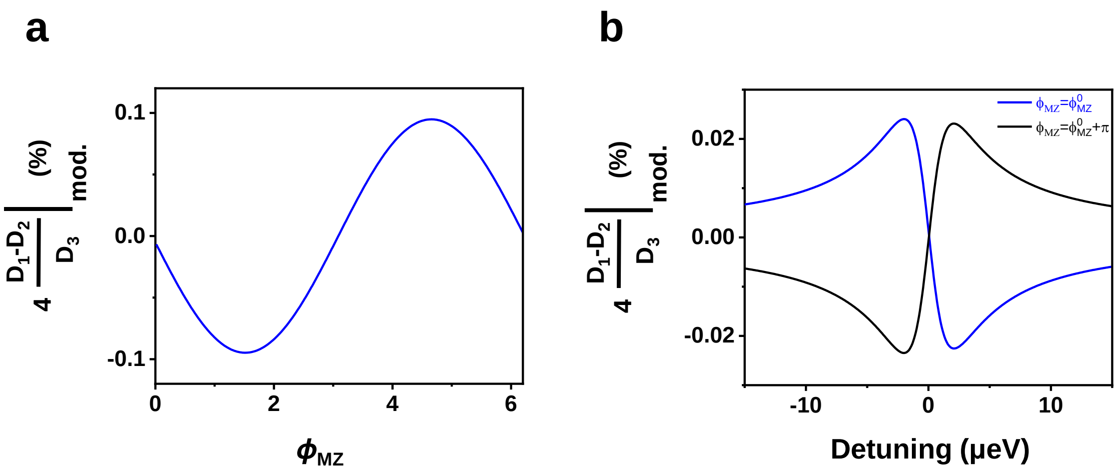

Supplementary Fig. 3: MZI phase dependence on the phase delay. a, Theoretical pump induced phase delay at 0 detuning as a function of the MZI phase (ϕ_{MZ}). **b**, Pump induced phase delay at the zero crossings of **a**. The first zero crossing ($\phi_{MZ}^{0} \cong 1rad$) is shown in black while the second zero crossing ($\phi_{MZ}^{0}+\pi$) is shown in blue.

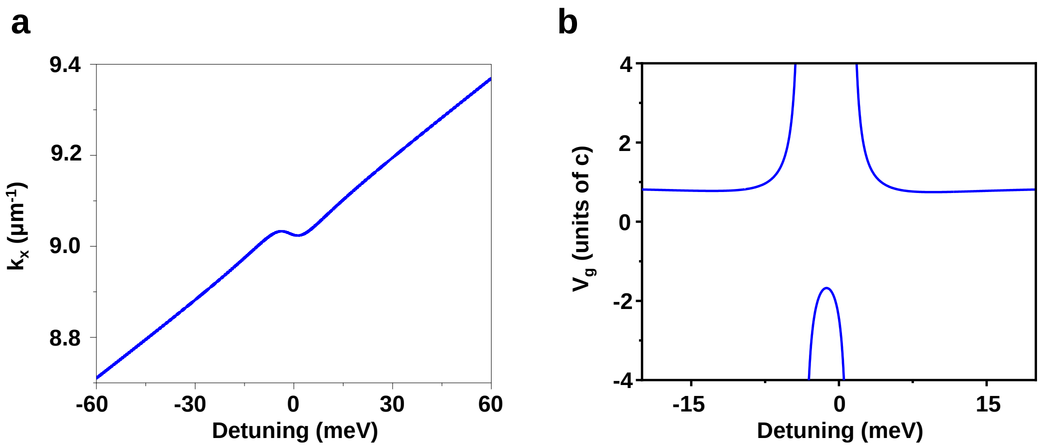

Supplementary Fig. 4. Theoretical E-SPP in absence of a pump. a, Dispersion relation of the E-SPP in absence of a pump. **b,** Group velocity of the E-SPP as a function of the detuning from the exciton resonance in absence of a pump.

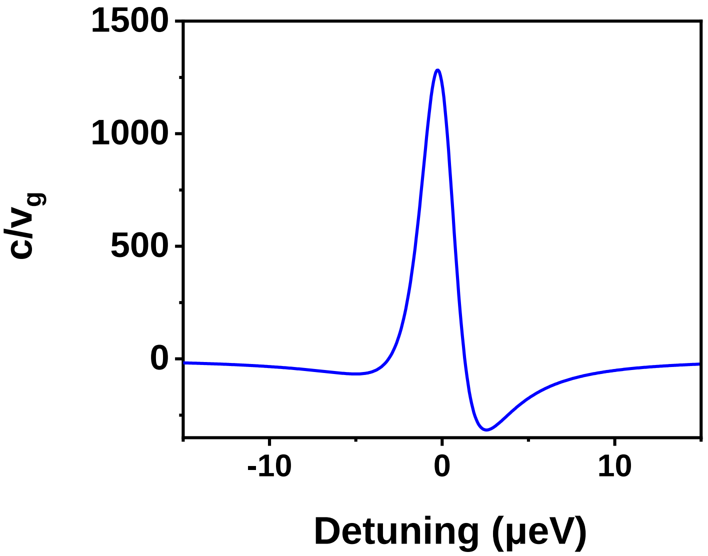

Supplementary Fig. 5: Detuning dependence of the slowdown. Theoretical plot of the slowdown as a function of the pump and probe detuning.

Supplementary Note 1. Theory of E-SPP population pulsation

Our theory is based on the formalism given in Ref. [1] and extended along the lines of Ref. [2]. In Ref. [1], the third-order nonlinear response of a few-level system was developed and a detailed analogy to many-particle theories was given.

Due to the optical selection rules, there is one ground state (labeled '1') coupled to two (optically active) one-exciton states (labeled '2'), which are further labeled by the exciton spin quantum number, 2_+ and 2_-, respectively. These two states, '+' and '-', correspond to excitons created by an optical interband transitions with a sum of the electron and hole angular momenta of +1 and -1 (in units of $\hbar$), respectively.

As discussed in Sec. IV of Ref. [1], going beyond the coherent time scale requires the introduction of incoherent (or long-lived) exciton occupations, which means that the level of the one-exciton state does not only represent the exciton with zero center-of-mass momentum (the 'coherent' exciton), but the entire continuum of one-exciton states with different center-of-mass momenta. Hence, the total exciton population is a sum of the coherent population and the incoherent (long-lived) population, see Eq. 17 of Ref. [1]:

$$\rho^{(2)}_{2_\pm 2_\pm}(t) = \left|\rho^{(1)}_{2_\pm 1}(t)\right|^2 \pm f^{inc(2)}_{\pm\pm} \tag{1}$$

Here, $\rho^{(1)}_{2_\pm 1}(t)$ is the density-matrix element for the transition from the ground state (1) to the one-exciton state ($2_\pm$), $\rho^{(2)}_{2_\pm 2_\pm}(t)$ is the total exciton population in second order, and $f^{inc(2)}_{\pm\pm}$ the incoherent part of the total exciton population. We will omit for now the superscripts (1) and (2) indicating the first and second order in the applied field, respectively.

In Ref. [1], the incoherent exciton population $f^{inc}_{\pm\pm}$ decays to the ground state at a rate γ_f. Reference [1] also allows for the decay of the exciton spin coherence, $f^{inc}_{\pm}$ at rate $\gamma_{2_+2_-}$, but it does not account for a different decay rate of the exciton spin imbalance, i.e. the difference between the

exciton populations in states 2_+ and 2_-, respectively. However, in Ref. [2] it was shown that allowing the average and difference of the long-lived state populations to decay with different rates led to signatures in the nonlinear optical response that had been observed experimentally, and that were interpreted as population pulsations. We therefore use the strategy of [2] and extend the formalism of [1] to allow for different decay rates of the total exciton population

$$\rho_{tot}(t) = \rho_{2_+2_+}(t) + \rho_{2_-2_-}(t) \tag{2}$$

and the population difference, representing the spin imbalance

$$\rho_{diff}(t) = \rho_{2_+2_+}(t) - \rho_{2_-2_-}(t) \tag{3}$$

We then assume that each of the two spin states either decays to the ground state (at rate γ_f) or decays through scattering to the opposite spin (at rate γ_d), and grows through scattering originating from the opposite spin

$$\hbar \frac{d}{dt}\rho_{2_+2_+}(t)\bigg|_{decay} = -(\gamma_f + \gamma_d)\rho_{2_+2_+}(t) + \gamma_d \rho_{2_-2_-}(t) \tag{4}$$

and

$$\hbar \frac{d}{dt}\rho_{2_-2_-}(t)\bigg|_{decay} = -(\gamma_f + \gamma_d)\rho_{2_-2_-}(t) + \gamma_d \rho_{2_+2_+}(t) \tag{5}$$

For the decay of the total and difference population we then have

$$\hbar \frac{d}{dt}\rho_{tot}(t)\Big|_{decay} = -\gamma_f \rho_{tot}(t) \tag{6}$$

and

$$\hbar \frac{d}{dt}\rho_{diff}(t)\Big|_{decay} = -\left(\gamma_f + 2\gamma_d\right)\rho_{diff}(t) \tag{7}$$

We then proceed along the lines of Ref. [1] to obtain the response in probe direction, assuming steady state for both pump

$$\Omega_{\pm}^{p}(t) = \Omega_{\pm}^{p} e^{-i\omega_p t} \tag{8}$$

and probe (or signal denoted by superscript s)

$$\Omega_{\pm}^{s}(t) = \Omega_{\pm}^{s} e^{-i\omega_s t} \tag{9}$$

The subscripts/superscripts, p and s, used in the Supplementary Information correspond to pp and pr, respectively, in the main text. Written in terms of electric field amplitudes, the Rabi frequencies are $\Omega_{\pm}^{a}(t) = \mu_{\pm} E_{\pm}^{a}(t)$, where $E_{\pm}^{a}(t)$ are the circularly polarized components of pump and probe fields, a = p,s, and $\mu = -er_{cv}$ is the interband dipole matrix element (see [3] for more details of

interband matrix elements), with e being the electron charge in vacuum. Furthermore, we use the first-order exciton susceptibility

$$\chi_{\pm}^{(1)}(\omega) = -\frac{|\varphi_{1s}(r=0)|^2 \mu_{\pm}^2}{\hbar\omega - \varepsilon_{1s} + i\gamma_2} \tag{10}$$

where $\varphi_{1s}(r=0)$ is the 1s exciton wave function at zero relative coordinate, ε_{1s} the 1s exciton energy, and γ_2 the exciton dephasing. Here and in the following, the susceptibilities are 2D susceptibilities with units of length, where we assume the corresponding 3D susceptibilities to be of the form $\chi^{(3D)}(\omega, z) = \frac{1}{L_z}\chi^{(2D)}(\omega)$ in the interval $0 \le z \le L_z$ and zero else, with L_z being the layer thickness. The interband dipole matrix element is the same in both spin sub-systems, making $\mu_{\pm} \equiv \mu$ and $\chi_{\pm}^{(1)}(\omega) \equiv \chi^{(1)}(\omega)$ independent of the subscript ±. Here, we have restored the superscript (1) to indicate first-order (in the applied field) response.

For the incoherent populations, we obtain, after some algebra, and in particular after using Eq. (19) of [1],

$$\begin{aligned} f_{\pm\pm}^{inc}(t) = \frac{1}{|\varphi_{1s}(r=0)|^4 \mu^2} \{ & A_{\pm} \left|\chi^{(1)}(\omega_p)\right|^2 \left|E_{+}^{p}(t)\right|^2 \\ & + A_{\mp} \left|\chi^{(1)}(\omega_p)\right|^2 |E_{-}^{p}(t)|^2 \\ & + B_{\pm} \chi^{(1)}(\omega_s) \chi^{(1)*}(\omega_p) E_{+}^{s}(t) E_{+}^{p*}(t) \\ & + B_{\mp} \chi^{(1)}(\omega_s) \chi^{(1)*}(\omega_p) E_{-}^{s}(t) E_{-}^{p*}(t) \} \end{aligned} \tag{11}$$

where

$$A_{\pm} = \frac{1}{2}\left(\frac{2\gamma_2 - \gamma_f}{\gamma_f} \pm \frac{2\gamma_2 - \widetilde{\gamma_d}}{\widetilde{\gamma_d}}\right) \tag{12}$$

and

$$B_{\pm} = \frac{1}{2}\left(\frac{2\gamma_2 - \gamma_f}{\gamma_f - i\Delta} \pm \frac{2\gamma_2 - \widetilde{\gamma_d}}{\widetilde{\gamma_d} - i\Delta}\right) \tag{13}$$

Here, $\widetilde{\gamma_d} = \gamma_f + 2\gamma_d$, and $\Delta = \omega_s - \omega_p$ is the detuning of the probe relative to the pump. After some further algebra, we obtain the following expressions for the third-order susceptibility, where we restrict ourselves to the phase-space filling process determined by the dimensionless phase-space filling factor v, which is of order one, and in the numerical computations we take $v = 1$.

The cross-circular response for a '+' polarized pump and a '-' polarized probe contains only A_- and thus no narrow resonance:

$$\Delta\chi_{-+}^{cross-circ}(\omega_s) = -2v\frac{\mu}{|\varphi_{1s}(r=0)|^2}\chi^{(1)}(\omega_s) \times A_-\left|\chi^{(1)}(\omega_p)\right|^2\left|E_+^p\right|^2 \tag{14}$$

The co-circular response for '+' polarized pump and probe fields contains B_+, and therefore one narrow resonance with width $\widetilde{\gamma_d} = \gamma_f + 2\gamma_d$, and one ultranarrow resonance with width γ_f:

$$\Delta\chi_{++}^{co-circ}(\omega_s) = -2v\frac{\mu}{|\varphi_{1s}(r=0)|^2}\chi^{(1)}(\omega_s) \times [(1+A_+)\left|\chi^{(1)}(\omega_p)\right|^2 + (1+B_+)\chi^{(1)}(\omega_s)\chi^{(1)*}(\omega_p)]\left|E_+^p\right|^2 \tag{15}$$

The co-linear (both fields 'y' polarized) and the cross-linear (pump 'y' polarized and probe 'x' polarized) responses are then given by

$$\Delta\chi^{co-linear/cross-linear}(\omega_s) = -2v\frac{\mu}{|\varphi_{1s}(r=0)|^2}\chi^{(1)}(\omega_s) \qquad (16)$$

$$\times [(1 + A_+ + A_-)\left|\chi^{(1)}(\omega_p)\right|^2 + (1 + B_+ \pm B_-)\chi^{(1)}(\omega_s)\chi^{(1)*}(\omega_p)]\frac{1}{2}\left|E_y^p\right|^2$$

where the upper sign (B_+ + B_-) is for co-linear, and the lower sign (B_+- B_-) for cross-linear. We see immediately that the co-linear case, where we have $B_+ + B_- = \frac{2\gamma_2-\gamma_f}{\gamma_f-i\Delta}$ has only the ultranarrow resonance, while the cross-linear case, with $B_+ - B_- = \frac{2\gamma_2-\widetilde{\gamma_d}}{\widetilde{\gamma_d}-i\Delta}$ has only the narrow resonance. In the numerical evaluation, we use $\gamma_f = 0.002$meV, $\gamma_d = 0.02$meV, $\gamma_2 = 6.448$meV, $\varphi_{1s}(r = 0) = 0.79cm^{-1}$, $r_{cv} = 0.3$nm.

From the knowledge of the susceptibility without the pump, $\chi^{without-pump}(\omega) = \chi^{(1)}(\omega)$ and that in the presence of the pump, $\chi^{with-pump}(\omega) = \chi^{(1)}(\omega) + \Delta\chi(\omega)$, we obtain the differential reflection (DR/R) and differential E-SPP transmission (DT/T) using the configuration seen in Fig. 2 of the main text. For the E-SPP differential transmission, we use the formalism presented in [4] and express the wavevector k_x of the E-SPP in terms of the susceptibility, to obtain the transmission with and without pump. For the former, where the DR/R signal relates to an all-optical configuration seen in Fig. 2b, we use a transfer matrix method [5] specialized to a delta-sheet approximation (i.e. $L_z \to 0$) relating the amplitudes of the right-traveling (R_+) and left-traveling (R_-) waves on the right, to those on the left, (L_+, L_-), via

$$\begin{pmatrix} R_+ \\ R_- \end{pmatrix} = \begin{pmatrix} \Delta_+ - i\beta & \Delta_- - i\beta \\ \Delta_- + i\beta & \Delta_+ + i\beta \end{pmatrix}\begin{pmatrix} L_+ \\ L_- \end{pmatrix} \qquad (17)$$

with the beam incident from the left. Here, $\beta = -2\pi\frac{\omega}{cn_R}\chi(\omega)$ where n_R (n_L) is the refractive index on the right (left) of the layer, and $\Delta_{\pm} = (n_R + n_L)/(2n_R)$. The reflectivity is then $R = |L_-/L_+|^2$, assuming no incident beam on the right ($R_- = 0$).

Supplementary Note 2. Calculation of the group velocity

In order to extract the group velocity from the slope of the (D1-D2) interference signal, we first consider the two fields from a balanced Mach-Zehnder interferometer (MZI) where one arm (here denoted as arm 2) has a piezoelectric translator (PZT) giving rise to a phase delay of $\varphi_{p.z.}$, and the sample material giving rise to a phase delay kL in arm 1. After combining the light from the two arms in with the second beam splitter, the two outputs of the second beam splitter are

$$E_1^{out} = i\frac{1}{2}E_{pr}\left(e^{i\phi_1} + e^{i\phi_2}\right) \tag{18}$$

$$E_2^{out} = \frac{1}{2}E_{pr}\left(e^{i\phi_1} - e^{i\phi_2}\right) \tag{19}$$

where $\phi_1 = \phi_{1,0} + kL$, with $\phi_{1,0} =$ total phase in arm 1 excluding E-SPP, $\phi_2 = \phi_{2,0} + \phi_{p.z.}$, with $\phi_{2,0} =$ total phase in arm 2 excluding piezo state, and $k = n(\omega)\omega/c + i\alpha(\omega)/2$.

We then define the signal coming from one of the outputs of the MZI,

$$D_1 = \eta|E_1^{out}|^2 = \frac{1}{4}\eta\left|E_{pr}\right|^2 e^{-\alpha L}\left|1 + e^{i(\phi_2-\phi_1)}\right|^2 \tag{20}$$

with

$$e^{i(\phi_2-\phi_1)} = e^{i(\phi_2-\phi_{1,0})}e^{-in\frac{\omega}{c}L}e^{\alpha\frac{L}{2}} \tag{21}$$

where η is a detector constant, and the frequency arguments of the refractive index and the absorption coefficient are suppressed. We then rewrite the signals coming from the two outputs of the MZI as

$$D_1 = \frac{1}{4}\eta\left|E_{pr}\right|^2 e^{-\alpha L}[1 + e^{\alpha L} + 2e^{\frac{1}{2}\alpha L}\cos\left(\phi_2 - \phi_{1,0} - n\frac{\omega}{c}L\right) \tag{22}$$

$$D_2 = \frac{1}{4}\eta\left|E_{pr}\right|^2 e^{-\alpha L}[1 + e^{\alpha L} - 2e^{\frac{1}{2}\alpha L}\cos\left(\phi_2 - \phi_{1,0} - n\frac{\omega}{c}L\right) \tag{23}$$

We also define an additional signal for the beam before it gets split by the beam splitter

$$D_3 = \eta\left|E_{pr}\right|^2 e^{-\alpha L} \tag{24}$$

Subtracting D_2 from D_1 gives us only one term that is related to the refractive index of the material in one arm of the MZI (also seen in [6]),

$$D_1 - D_2 = \eta\left|E_{pr}\right|^2 e^{-\frac{1}{2}\alpha L} cos\left(\phi_{MZ} - n\frac{\omega}{c}L\right) \tag{25}$$

where $\phi_{MZ} = \varphi_{2,0} - \varphi_{1,0} + \varphi_{p.z.}$ and normalization by the D_3 signal yields the final expression for the MZI signal

$$F = \frac{D_1 - D_2}{D_3} = e^{\frac{1}{2}\alpha L} \cos\left(\phi_{MZ} - n\frac{\omega}{c}L\right) \tag{26}$$

The phase delay's dependence on the Mach Zehnder phase is shown in Fig. S3.

We then adjust the MZI phase ϕ_{MZ} such that at $\omega=\omega_0$ the MZI signal vanishes, F=0. We denote this MZ phase by ϕ_0:

$$\phi_0 - n(\omega_0)\frac{\omega_0}{c}L = \frac{\pi}{2} \tag{27}$$

Now, the frequency dependence of the signal is given by

$$F = e^{\frac{1}{2}\alpha L}\sin\,(n(\omega)\frac{\omega}{c}L - n(\omega_0)\frac{\omega_0}{c}L) \tag{28}$$

and in the vicinity of ω_0 we have (with the sine-function approximated by its argument)

$$F(\omega) \cong e^{\frac{1}{2}\alpha L}(\omega - \omega_0)\frac{d}{d\omega}n(\omega)\frac{\omega}{c}L\Big|_{\omega_0} + O((\omega - \omega_0)^2) \tag{29}$$

where we neglect the frequency dependence of the absorption coefficient close to ω_0.

We now define the slowdown to be the inverse group velocity at ω_0 in units of the speed of light, $S = {}^{c}/_{v_g} = \frac{d}{d\omega}n(\omega)\omega|_{\omega_0}$.

We then treat $n(\omega)$ as $n(\omega) = n^{(1)}(\omega) + \Delta n(\omega)$ where $n^{(1)}(\omega)$ is the index of refraction in the absence of the pump and $\Delta n(\omega)$ is the change in the index of refraction due to the pump, so that

$S = S_0 + \Delta S$ where $S_0 = \frac{d}{d\omega} n^{(1)}(\omega)\omega|_{\omega_0}$ is the slowdown without the pump and $\Delta S = \frac{d}{d\omega} \Delta n(\omega)\omega|_{\omega_0}$ is the pump-induced change of the slowdown. Now the frequency derivative of the detector signal evaluated at ω_0 becomes $\frac{d}{d\omega} F(\omega)|_{\omega_0} = e^{\frac{1}{2}\alpha L} \frac{L}{c}(S_0 + \Delta S)$. Which then implies that $S = e^{-\frac{1}{2}\alpha L} \frac{c}{L} \frac{d}{d\omega} \frac{D_1 - D_2}{D_3}|_{\omega_0}$.

Supplementary Note 3. Extracting nonlinear response from measured signal.

In our DT measurements, we use a double modulation technique to isolate the nonlinear response. That is, we use a lock-in amplifier measuring at the difference of the modulation frequencies between the pump and the probe which is only a fraction of the total nonlinear signal.

The intensities of our pump (subscript p) and probe (subscript pr) beams can be written as

$$I_{pr} \propto |E_{pr}|^2 = \frac{1}{2}|E_{pr,0}|^2\left(1 + \cos(\Omega_{pr} t)\right) \tag{30}$$

$$I_p \propto |E_p|^2 = \frac{1}{2}|E_{p,0}|^2\left(1 + \cos(\Omega_p t)\right) \tag{31}$$

where $E_{pr,0}, E_{p,0}$ are the electric field amplitudes, Ω_{pr}, Ω_p are the intensity modulation frequencies and t is time. Since we restrict ourselves to the lowest-order nonlinear response regime, the so-called $\chi^{(3)}$ regime, the change of the refractive index is proportional to the pump intensity. With the temporal modulation of the pump, the modulated pump-induced change of the refractive index becomes

$$\Delta n(\omega, t) = \Delta n(\omega)\ \frac{1}{2}\left(1 + \cos(\Omega_p t)\right) \tag{32}$$

Taking into account the pump and probe modulation, and noting that only ΔS is modulated at the pump modulation frequency, the $D_1 - D_2$ signal becomes

$$D_1 - D_2 \approx \eta \left|E_{pr,0}\right|^2 \frac{1}{2}\left(1 + \cos\left(\Omega_{pr} t\right)\right) e^{-\frac{1}{2}\alpha L}\left(S_0 + \Delta S \frac{1}{2}\left(1 + \cos\left(\Omega_p t\right)\right)\right) \tag{33}$$

and the D_3 signal is

$$D_3 = \eta \left|E_{pr,0}\right|^2 \frac{1}{2}\left(1 + \cos\left(\Omega_{pr} t\right)\right) e^{-\alpha L} \tag{34}$$

where α is the material absorption coefficient, L is the material's length, and S_0 and ΔS correspond to the linear and nonlinear slowdown factors defined in Supplementary Note 2.

In our measurements, we only retain the component of $D_1 - D_2$ oscillating at the difference frequency $\Omega_p - \Omega_{pr}$, and the dc-component (zero frequency) of D_3. Hence, in our measurements the contribution of S_0 drops out (it has no pump-induced modulation), and only the ΔS contribution is retained. Using

$$\begin{aligned} &\frac{1}{2}\left(1 + \cos\left(\Omega_{pr} t\right)\right) \frac{1}{2}\left(1 + \cos\left(\Omega_p t\right)\right) \\ &\qquad = \frac{1}{8}\cos\left(\left(\Omega_p - \Omega_{pr}\right) t\right) + \mathrm{non-difference-frequency\ terms} \end{aligned} \tag{35}$$

which means that retaining only the difference-frequency component effectively multiplies the ΔS term by a factor 1/8. Similarly, the DC-component of D_3 is $\eta \left|E_{pr,0}\right|^2 \frac{1}{2} e^{-\alpha L}$, which means that

dividing by the DC-component of D_3 yields a factor of $1/(\frac{1}{2})$. We can then write the modulated measured signal as

$$4\,\frac{D_1-D_2}{D_3}\bigg|_{mod} = e^{\frac{1}{2}\alpha L}\,\frac{L}{c}\,\Delta S. \tag{36}$$

This factor of 4 is included in all of our nonlinear plots as both the DT/T and phase delay measurements use the same modulation scheme.

Supplementary References

1. H. Kwong, N., Rumyantsev, I., Binder, R. & Smirl, A. L. Relation between phenomenological few-level models and microscopic theories of the nonlinear optical response of semiconductor quantum wells. *Physical Review B* **72**, 235312 (2005).
2. Schaibley, J. R. *et al.* Population Pulsation Resonances of Excitons in Monolayer $MoSe_2$ with Sub-1 μeV Linewidths. *Physical Review Letters* **114**, 137402 (2015).
3. Gu, B., Kwong, N. H. & Binder, R. Relation between the interband dipole and momentum matrix elements in semiconductors. *Physical Review B* **87**, 125301 (2013).
4. Klein, M. *et al.* 2D semiconductor nonlinear plasmonic modulators. *Nature Communications* **10:3264**, (2019).
5. Nenno, D. M. *et al.* Modification of spintronic terahertz emitter performance through defect engineering. *Scientific Reports* **9:13348**, (2019).
6. Ku, P.-C. *et al.* Slow light in semiconductor quantum wells. *Optics Letters* **29**, 2291 (2004).
